# Effect of Postoperative Anesthesiologists’ Single Visit on Patient Satisfaction: A Hospital-Based Non-Randomized Study

**DOI:** 10.7759/cureus.34518

**Published:** 2023-02-01

**Authors:** Anjum Goth, Habib Md R Karim, Mohd Yunus, Raunaq Chakraborty, Samarjit Dey, Prithwis Bhattacharyya

**Affiliations:** 1 Anesthesiology, University Hospital of North Tees, Stockton-On-Tees, GBR; 2 Anesthesiology, Critical Care, and Pain Medicine, All India Institute of Medical Sciences, Raipur, IND; 3 Emergency Medicine and Trauma, All India Institute of Medical Sciences, Bhopal, IND; 4 Anesthesiology, All India Institute of Medical Sciences, Mangalagiri, IND; 5 Anesthesiology and Critical Care, North Eastern Indira Gandhi Regional Institute of Health & Medical Sciences (NEIGRIHMS), Shillong, IND

**Keywords:** patient’s satisfaction, anesthesiologists, postoperative care, personal satisfaction, continuity of patient care, anesthesia

## Abstract

Background

Continuity of personal care by the anesthesiologist is crucial for patient satisfaction. Over and above the consultation and service in the preoperative area, intraoperative care, and post-anesthesia care unit, anesthesia services frequently incorporate a pre-anesthesia evaluation clinic and a preoperative visit in the inpatient ward for their services, which helps with rapport building. However, routine post-anesthesia visits in the inpatient ward by the anesthesiologist are infrequent, causing a break in the continuity of care. The effect of such a routine post-operative visit by anesthesiologists has been tested only rarely in the Indian population. The present study aimed to evaluate the impact of a single postoperative visit by the same anesthesiologist (continuity of care) on patient satisfaction and compare it with a postoperative visit by another anesthesiologist and no postoperative visit.

Methods

After institutional ethical committee approval, 276 American Society of Anesthesiologists physical status (ASA PS) I and II, consenting, elective surgical inpatients older than 16 years were enrolled in a tertiary care teaching hospital from January 2015- September 2016. Consecutive patients were allocated into three groups based on the postoperative visit (i.e., group A: by the same anesthesiologist; group B: another anesthesiologist; and group C: no visit). Data related to patients’ satisfaction were collected in a pretested questionnaire. Chi-Square and Analysis of Variance (ANOVA) were applied to analyze the data and compare among the groups; a p < 0.05 was considered statistically significant.

Results

The mean age of the entire cohort was 38.1 years, comprising 39.9% men. Demographic, socioeconomic, and educational statuses were similar in all groups (p >0.05). The percentages of patient satisfaction were 61.47%, 51.52%, and 38.5% in groups A, B, and C, respectively (p=0.0001). Satisfaction with the fulfillment of "continuity of personal care" was the highest for group A (69.35%), which was significantly higher than group B (43.69%) and group C (35.65%). Group C had the lowest fulfillment of patient expectations and was significantly less satisfied than even group B (p=0.02).

Conclusion

Continuity of anesthesia care with the addition of routine postoperative visits had the highest positive impact on patient satisfaction. Even a single postoperative visit by the anesthesiologist significantly increased the patients’ satisfaction.

## Introduction

Patient satisfaction is an essential and commonly used indicator for measuring healthcare quality. It affects patient retention, malpractice claims, and clinical outcomes. Patient-centered care is one of the aims of a quality healthcare system set by the Institute of Medicine, Washington [1]. In recent years, there has been an increased emphasis on the role of anesthesiologists as perioperative physicians and educators, and the anesthesiologist's service has spread to preoperative, postoperative, and beyond [2,3].

Continuity of personal care by an anesthesiologist has been defined as the presence of the same anesthesiologist providing pre-operative evaluation, informed consent, performing anesthesia, and visiting the patient after an operation [4,5]. It has been described as one of the crucial factors in patient satisfaction with anesthesia care [4,5]. A very high prevalence (77%) of dissatisfaction with the perception of not being visited by an attending anesthesiologist after an operation has been reported [5]. However, data in this context from India and other developing countries is limited and must also be available. Further, how many postoperative visits are required to improve patient satisfaction is also unknown. The present study was designed to evaluate the impact of a single postoperative visit by the same anesthesiologist (continuity of personal care) on patient satisfaction. We also aimed to compare the satisfaction or dissatisfaction with continuity of personal care with a postoperative visit by another anesthesiologist and no postoperative visit. The study results are likely to improve perioperative care plans in the future.

## Materials and methods

After approval from the institute's Ethical Committee, the present non-randomized control study was conducted from January 2015 through September 2016 at the North Eastern Indira Gandhi Regional Institute of Health and Medical Sciences (NEIGRIHMS), a tertiary care academic institute in Shillong, India. Elective surgical patients aged 16 years or more of either sex, belonging to American Society of Anesthesiologists (ASA) I and II physical status, who underwent surgery as inpatients and had reasonable (can read and understand) knowledge of the local language or Hindi or English, were approached for consent and enrollment in the study. Patients who required a postoperative anesthesiologist's visit or follow-up visit for health or comorbidity-related reasons or anesthesia-related complications on the first postoperative day were excluded.

The study was conducted in a tertiary care academic center where anesthesia service routinely comprises evaluating a patient in the pre-anesthesia evaluation clinic, providing necessary information to the patient, and discussing the anesthesia techniques and possible complications and management. Once the anesthesia team clears the patient for elective surgery with risk stratification and advice, the patient gets admitted to the inpatient ward, usually the day before the scheduled surgery. A resident of the anesthesia team re-visits the patient in the inpatient ward, re-evaluates, discusses, clarifies the doubts, if any, and alleviates the apprehension; informed written consent for anesthesia is also obtained. On the day of surgery, the patient is shifted to the preoperative area by the hospital attendant and nursing officer taking care of him or her in the ward. The anesthesia resident and consultant meet the patient and prepare the patient for surgery. A resident, along with the operation theater attendant and nursing officer, shifts the patient inside the operating room, and anesthesia services for intraoperative care are provided. Post reversal and extubation, the anesthesia team shifts the patient to the post-anesthesia care unit, where the patient is cared for under the direct supervision of the anesthesia team till he or she is found suitable to be shifted back to the ward. However, routine post-anesthesia visits of the patients by the anesthesiologist in the ward were not practiced except for the acute pain service team visit, which led to the discontinuation of the services to be addressed as continuity of anesthesia care as defined by Saal et al. [4,5]. Although the hospital also has a dedicated team comprising anesthesiologists, residents, nursing officers, and volunteers for social outreach, the service was meant for palliative care, and none of the enrolled patients were in this category. Based on this existing workflow for anesthesia services, three groups of patients were enrolled based on the postoperative anesthesiologist's visit: group A, in which the same anesthesiologist who conducted anesthesia also visited the patient; group B, in which another anesthesiologist who was not involved in the delivery of anesthesia visited the patient; and group 3, the control group, which did not receive a postoperative visit. We considered group A to be the continuity of personal care, as it fulfilled the criteria laid out by Saal et al. [4,5].

A pre-tested questionnaire derived from a previously published article [5] and ASA online material [6] on the same subject was used as a study tool or instrument. The English version of the questionnaire was translated into local and Hindi languages using the modified World Health Organization method [7]. This final questionnaire was tested as a pilot questionnaire for one month for its applicability in our setup before undertaking this prospective study and was adapted as required. The questionnaire questions were grouped into eight categories: information and involvement in decision-making; respect and confidence; care in the recovery room; continuity of personal care; pain management; attention; privacy; and overall quality of care, as per the categories described in the base articles [5,6] and presented in Annexure 1. The on-duty nursing officers handed the questionnaire to the patient after explaining it in their language. On-duty staff nurses of the respective surgical department collected the questionnaire, which the investigator then collected on the day of discharge from the patient's file.

The scoring was also adapted from the base study by Saal et al. [5]. If a question was not answered 100% positively (i.e., "yes, definitely," "completely satisfied," etc.) but partially negatively (i.e., "yes, somewhat," "partially satisfied," etc.), or entirely negatively, it was taken as a deficit (dissatisfaction). The sum of all the deficits in the problem questions led to the problem score for each of the eight dimensions. The mean of the problem scores for all the problem questions formed the "total problem score." The continuity of personal care by the anesthesiologist, in combination with the importance of a postoperative visit for the patients, was also evaluated. It was defined as a problem if the patients considered continuity of personal care by the attending anesthesiologist essential and did not receive a postoperative visit or remember such a visit [5]. However, it was not considered a negative response if the patients did not consider such a visit important and nobody visited them in the postoperative period. We also measured the "negative patient response," also called the "problem score," created by the perception of not being visited after an operation by the attending anesthesiologist, and the effect of this problem score on the dimension "continuity of personal care by the anesthesiologist." The overall dissatisfaction with anesthesia care was expressed as the "Total Problem Score" [5].

The present study sample size was calculated based on a previous study with a prevalence of dissatisfaction of 8% versus 28% for the perception of not being visited by an attending anesthesiologist after an operation versus a visit by a different anesthesiologist [6]. The sample was calculated using the online tool OpenEpi, which provides open-source epidemiologic statistics for public health [8]. The study's precision (1- α) was set at 95% and power (1-β) at 80% with an exposure to a non-exposed ratio of 1, which gave 42 participants for each group. Taking 20% of the sample as non-responders and a design effect of 1.8, the sample size per group was 92. Consecutive sampling with sequential patient allocation was used. Although the study could have been designed as a randomized one, due to some technical difficulties, it was not done at the time of protocol formation. A total of 276 (92 x 3) consented patients were recruited in the study, and consecutive eligible patients were assigned to one of the three groups (viz. patient one, four, seven, and so on to one group; two, five, eight and so on in another group; and three, six, nine, and so on to the other group).

Statistical analysis of the data was performed using Statistical Package for the Social Sciences (SPSS) version 20 software (IBM Corporation, Armonk, New York, United States). The comparisons of the total problem score among the three groups were done. Patients' clinical profiles and other categorical data are presented in absolute numbers, percentage scales, or with central dispersion as required and were statistically analyzed by the Chi-square test for qualitative variables and the one-way Analysis of Variance (ANOVA) test for quantitative variables; p <0.05 was considered significant.

## Results

The 276 patients approached for participation consented to the study, and none dropped out. Out of this, 39.86% of participants were male and 60.14% were female. The mean ± standard deviation (SD) age was 38.11 ± 13.38 years. The socio-demographic parameters were comparable in all three groups (p >0.05). The ASA physical class and surgical department-wise distribution of patients were also comparable without statistically significant differences (p >0.05); however, the groups were significantly different in terms of the distribution of the sexes (Table 1).

**Table 1:** Demographic, educational, socioeconomic, and surgical discipline-wise distribution and comparison of the groups. SD – standard deviation; ASA PS – American Society of Anesthesiologist physical status; IQR – interquartile range

Parameters	Number (%) (N for each group: 92)			p-value
	Group A	Group B	Group C	
Age (year) mean ± SD	38.71 ± 13.62	36.33 ± 11.46	39.28 ± 14.79	0.284
Male	42 (45.65)	27 (29.35)	41 (44.57)	0.041
Female	50 (54.35)	65 (70.65)	51 (55.43)	0.041
ASA PS median (IQR)	1 (1-1)	1 (2-1)	1 (1-1)	0.261
ASA PS Class I	73 (79.35)	67 (72.83)	76 (82.61)	
ASA PS Class II	19 (20.65)	25 (27.17)	16 (17.39)	
Economic status: lower class	1 (1.09)	5 (5.43)	6 (6.52)	0.386
Economic status: lower middle class	23 (25.00)	20 (21.74)	19 (20.65)	
Economic status: middle class	59 (64.13)	59 (64.13)	53 (57.61)	
Economic status: upper middle	9 (9.78)	8 (8.70)	14 (15.22)	
Education level: primary education	13 (14.13)	5 (5.43)	10 (10.87)	0.166
Education level: high school	17 (18.48)	22 (23.92)	11 (11.95)	
Education level: higher secondary	21 (22.83)	25 (27.17)	19 (20.65)	
Education level: graduate; postgraduate	33 (35.86) 8 (8.70)	36 (39.13) 4 (4.35)	40 (43.47) 10 (10.86)	
Ear, nose, and throat cases	7 (7.61)	8 (8.70)	8 (8.70)	0.196
General surgery cases	39 (42.39)	35 (38.04)	30 (32.61)	
Gynecology cases	10 (10.87)	24 (26.09)	18 (19.56)	
Neurosurgery cases	5 (5.43)	2 (2.17)	8 (8.70)	
Orthopedics	18 (19.57)	10 (10.87)	11 (11.95)	
Interventional radiology cases	0	0	1 (1.09)	
Urology cases	13 (14.13)	13 (14.13)	16 (17.39)	

The percentage of satisfaction with overall anesthetic care was highest in group A, patients who the same anesthesiologist visited during the postoperative period (61.47%), followed by group B, patients who another anesthesiologist visited during the postoperative period (51.52%), and lowest in group C, patients without any postoperative visit by an anesthesiologist (38.5%) (Table 2).

**Table 2:** Satisfaction with anesthesia services in different groups and their comparison SD – standard deviation; CI – confidence interval.

Group	Overall percentage of satisfaction with anesthetic care			Percentage of satisfaction, taking the perception of not being visited after the operation only as a problem score		
	Mean ± SD	95 % CI	p-value	Mean ± SD	95 % CI	p-value
C	38.5 ± 14.42	35.52-41.49	Reference	39.05 ± 14.53	36.05-42.06	Reference
B	51.52 ± 17.03	48.0 -55.06	< 0.0001	52.01 ± 17.03	48.49-55.54	< 0.0001
A	61.47 ± 17.95	57.75-65.19	< 0.0001	61.52 ± 17.74	57.85-65.19	< 0.0001

The differences between the three groups were significant (p = 0.02). The dissatisfaction levels were calculated to be 38.53%, 48.48%, and 61.5% in the three groups. Taking the perception of not being visited after the operation as a problem score only if such a visit was considered necessary, the percentage of satisfaction was 61.52%, 52.01%, and 39.05%, respectively, in groups A, B, and C (p=0.0001). The calculated dissatisfaction levels were 38.48%, 47.99%, and 60.95%, respectively.

Satisfaction with the fulfillment of "continuity of personal care by anesthesiologists" was also highest in group A (69.35%), lower in group B (43.69%), and least in group C (35.65%). The differences between groups A versus B and A versus C were statistically significant (Table 3).

**Table 3:** Satisfaction about the continuity of personal care in different groups and their comparison, taking group C (standard care) as the reference SD- standard deviation, CI- confidence interval.

Group	% of satisfaction in context to continuity of personal care		
	Mean ± SD	95 % CI	p-value
C	35.65 ± 23.87	30.71-40.59	Reference
B	43.69 ± 25.15	38.49-48.90	0.027
A	69.35 ± 20.85	65.03-73.67	< 0.0001
Group A versus group B			< 0.0001

## Discussion

Dedicated personal care, including postoperative visits, is one of the most important factors for patient satisfaction with anesthesia care [9]. The present study also showed similar findings. An anesthesiologist's postoperative visit effectively increased the patients' satisfaction. There is no doubt that anesthesiologists have an outstanding track record in improving safety and reducing avoidable harm in surgical patients. However, there is variation in mortality among hospitals for the same type of surgery, and a lot can be done [10]. The postoperative period is crucial, and the long-term impacts of short-term postoperative harm are increasingly recognized. Postoperative visits can contribute directly or indirectly to the advancement of the surgical outcome, which is considered an essential component of healthcare ergonomics [11]. Therefore, with the anesthesiologist as a perioperative physician and core member of surgical patient care, it is high time to focus intensely on the postoperative period.

Measuring satisfaction is challenging, and universally applicable guidelines, methods, and scores have yet to be available. The available scoring systems also suffer from various potential limitations [11]. A psychometric questionnaire uses multiple items to probe specific events or concerns that occurred in that experience, events that together determine patients' satisfaction with their dimension of care. If a global satisfaction rating is necessary, ratings of individual items can be summed or averaged [12]. The questionnaire used for the present study was taken from a study conducted on the same topic that had been psychometrically constructed and validated. In addition, the questionnaire was also pretested in our institute for ease of acceptance.

An Australian study found an overall low dissatisfaction rate (3.2%) [13]. However, it was recognized that patient responses might be modified by a desire to please hospital staff and thus may be an underrepresentation of the actual dissatisfaction level [14]. Recent studies show only moderate satisfaction (50%-75%) [15,16]. It has been suggested that patients may need to learn what to expect from their hospitalization experience to rate their satisfaction appropriately or only rate selected aspects of their care [17]. Keeping this in mind, we asked patients to rate their satisfaction with anesthesia care only when our service's outcomes were known and fresh in their minds. They were not asked to rate their entire hospitalization episode, for which various factors are important [18].

Regular post-anesthetic visits allow the detection of anesthesia-related complications and increase patient satisfaction. Nevertheless, data concerning the current practice of post-anesthetic visits in India and other developing countries need to be available. A German survey found that only 20% of patients (the median) were estimated to receive a routine postanesthetic visit. However, this number was higher in hospitals with a specific postanesthetic visit service (median: 65%) [19]. While 98% of all respondents believed that post-anesthetic visits improved the quality of their work, 38.0% of the survey respondents reported detecting perioperative complications intermittently during their visits [19]. A study found a strong relationship between patient dissatisfaction and poor postoperative pain control, moderate nausea, and static and dynamic severe pain [19]. These factors associated with dissatisfaction can be prevented or better treated with postoperative visits.

The present study found 61.47% and 51.52% satisfaction with a postoperative visit by the same and another anesthesiologist, respectively. Although the satisfaction was significantly higher than the patients without any postoperative anesthesiologist visit (38.5%), the satisfaction was relatively low compared to the previous studies [13,15,16]. An Austrian study found that the negative patient response created by the perception of not being visited after an operation by the attending anesthetist was 77.1% in a group of patients who were not at all visited postoperatively [5]. A recent study has shown that postoperative visits and pain management can improve satisfaction by 99% [20]. Although this finding echoes the present study, some studies showed that only two or more postoperative visits by an anesthesiologist improved patient satisfaction [21]. The present study showed that a single postoperative visit by the attending anesthesiologist (continuity of personal care) significantly increased satisfaction compared with no visit, even when compared with a visit by a different anesthesiologist.

Although the present study was prospective, it was a non-randomized, questionnaire-based study. Interviewer bias, a chain of communication (due to the language barrier), and a desire to please might have been present. Further, the present study is a single-center study, and a cultural difference might also impact the patient satisfaction results noted. Continuity of personal care has multiple components, and service starts right from attending the hospital to discharge with coordination among the team members. Our hospital anesthesia service already had a workflow of care from the outpatient department to the post-anesthesia care unit. We added a routine postoperative visit to term it "continuity of anesthesia services," as defined by Saal et al. [4,5]. However, it might not be the same for other hospitals, or even the concept of continuity of personal care might differ in different countries.

## Conclusions

Our study findings indicate that the overall satisfaction in elective surgical patients during the perioperative period and satisfaction with anesthesia services is only moderate, ranging from 37% to 70%. However, there is ample scope to improve it only by incorporating a postoperative visit, discussing the problem faced, and providing a solution, assurance, or pain management as required by an anesthesiologist. The present study result indicates that postoperative visits by the anesthesiologist can increase patients' satisfaction significantly compared to no visit. Continuity of personal care has the highest positive impact on patient satisfaction. Even a single postoperative visit by the anesthesiologist who met the patient preoperatively for evaluation in the outpatient department, obtained informed consent after discussing the anesthesia plan and possible effects of anesthesia, alleviated the apprehensions by clearing the doubts, and took care in the preoperative and intraoperative stages, was perceived highly positively by the patient.

## Appendices

Annexure-1: Questionnaire

Section A: Information/involvement in decision-making

1. Did the anesthesiologist encourage you to ask questions?

a.      Yes, definitely

b.      Yes, somewhat

c.       No

2. Did you ask the anesthesiologist any questions?

a.      Yes

b.      No

3. Did the anesthesiologist answer your questions?

a. Yes, definitely

b. Yes, somewhat

c. No

4. Do you feel that the decision to give general /spinal/epidural anesthesia is correct?

a. Yes, definitely

b. Yes, somewhat

c. No

5. To what degree were you satisfied with the amount of information from the anesthesia practitioners?

a.      Very dissatisfied

b.      Dissatisfied

c.      Slightly dissatisfied

d.      Slightly satisfied

e.      Satisfied

f.       Very satisfied

6. The information given to me by the anesthesia practitioners was understandable.

a.      Disagree very much

b.      Disagree moderately

c.      Disagree slightly

d.      Agree slightly

e.      Agree moderately

f.       Agree very much

7. The anesthesia practitioners explained how I would feel after anesthesia.

a.      Disagree very much

b.      Disagree moderately

c.      Disagree slightly

d.      Agree slightly

e.      Agree moderately

f.       Agree very much

Section B: Respect/confidence

8. Will you ask for the same anesthetist in the future, or are you not happy with the same doctor?

a.      Yes, definitely

b.      Yes, somewhat

c.       No

9. I recommend the anesthesia team to others in my family.

a.      Disagree very much

b.      Disagree moderately

c.      Disagree slightly

d.      Agree slightly

e.      Agree moderately

f.       Agree very much

Section C: Care in the recovery room

10. In the recovery, were you satisfied with the care by your anesthesiologist?

a.      Yes, definitely

b.      Yes, somewhat

c.      No

11. How satisfied were you with treating nausea and vomiting after the operation?

a.      Very dissatisfied

b.      Dissatisfied

c.      Slightly dissatisfied

d.      Slightly satisfied

e.      Satisfied

f.       Very satisfied

Section D: Continuity of personal care by an anesthetist

 12. Have you been informed by an anesthesiologist about your forthcoming anesthesia?

a. Yes

b. No

13. Did you know which anesthesiologist would conduct your anesthesia?

a. Yes

b. No

14. Did the same anesthesiologist who informed you conduct your anesthesia?

a. Yes

b. No

15. Who visited you after the operation? ------------------

16. How important do you consider receiving a postoperative visit by the same anesthesiologist who conducted your anesthesia?

a. Very important

b. Moderately important

c. Unimportant (including no opinion)

Section E: Pain management

17. Did you feel pain during or after the operation?

a. Yes

b. No

18. How do you rate your “pain (management) experience" in the postoperative period?

a. Excellent

b. Acceptable

c. It could have been better

d. Unacceptable 

19. Please identify which aspect of postoperative pain management you felt should have been attended to improve your postoperative pain experience.

----------------------------------------------------------------------------------------------------------------------------------------------------------------------

20. Did this anesthesiologist answer your questions and address your doubts about pain?

a. Yes, definitely

b. Yes, somewhat

c. No

21. Did talking with this anesthesiologist during this visit make you feel calmer and more relaxed?

a. Yes, definitely

b. Yes, somewhat

c. No

Section F: Privacy

22. To what degree did the anesthesiologist consider your privacy in the operating room and recovery area?

a.      Very dissatisfied

b.      Dissatisfied

c.      Slightly dissatisfied

d.      Slightly satisfied

e.      Satisfied

f.       Very satisfied

23. Was your anesthetist discrete in asking private questions and mindful of your comfort?

a. Yes, definitely

b. Yes, somewhat

c. No

Section G: Attention

24. To what degree did your anesthesia practitioners pay attention to your complaints, like pain and nausea?

a.      Very dissatisfied

b.      Dissatisfied

c.      Slightly dissatisfied

d.      Slightly satisfied

e.      Satisfied

f.       Very satisfied

25. To what degree was the anesthesia team willing to listen to your questions?

a.      Very dissatisfied

b.      Dissatisfied

c.      Slightly dissatisfied

d.      Slightly satisfied

e.      Satisfied

f.       Very satisfied

Section H: Overall quality of care

26. I would want to have the same anesthetic again.

a. Yes, definitely

b. Yes, somewhat

c. No

27. How satisfied were you with the care provided by the department of anesthesia?

a.      Very dissatisfied

b.      Dissatisfied

c.      Slightly dissatisfied

d.      Slightly satisfied

e.      Satisfied

f.       Very satisfied

28. Based on this experience, I have a good understanding of the role the anesthesiologist played in my surgery.

a.      Disagree very much

b.      Disagree moderately

c.       Disagree slightly

d.      Agree slightly

e.      Agree moderately

f.       Agree very much
